# Transactions of Twenty-seventh Session of Hom. Med. Soc. of Penna.

**Published:** 1892-07

**Authors:** 


TRANSACTIONS OF THE TWENTY-SEVENTH SESSION OF THE HOMŒOPATHIC MEDICAL SOCIETY OF THE STATE OF PENNSYLVANIA. Held at Pittsburgh, September 15th–17th, 1891.

This is a volume of some three hundred and forty pages and shows that the Society is not idle. Dr. August Korndœrfer is President, and Dr. J. Horner is Secretary. The different bureaus are well represented and all the papers up to the standard. W. S.

